# Pseudodominant Inheritance of Retinitis Pigmentosa Due to Mutations in the Phosphodiesterase 6B Gene: A Case Report

**DOI:** 10.7759/cureus.34933

**Published:** 2023-02-13

**Authors:** Andrea Robles Bocanegra, Javier Tato, Leonardo J Molina Thurin, Natalio Izquierdo, Armando L Oliver

**Affiliations:** 1 Ophthalmology, San Juan Bautista School of Medicine, Caguas, PRI; 2 Ophthalmology, Ponce Health Sciences University, Ponce, PRI; 3 Surgery, School of Medicine, Medical Sciences Campus, University of Puerto Rico, San Juan, PRI; 4 Ophthalmology, School of Medicine, Medical Sciences Campus, University of Puerto Rico, San Juan, USA

**Keywords:** retinitis pigmentosa, retinal dystrophies, inherited retinal disease, case report, bony spicules, pde6b variant, autosomal recessive retinitis pigmentosa

## Abstract

Mutations in the phosphodiesterase 6B *(PDE6B) *gene are a rare cause of autosomal recessive retinitis pigmentosa (arRP). We report on a non-consanguineous family with a pseudodominant inheritance of RP due to *PDE6B* mutations. We conducted a chart review of four members of a Puerto Rican family who underwent a comprehensive ophthalmic evaluation by at least one of the authors. The mutational screening was done using a genotyping microarray provided by Invitae Corporation, using next-generation sequencing (NGS) technology. Genomic DNA obtained from saliva samples is enriched for targeted regions using a hybridization-based protocol and sequenced using Illumina technology. A descriptive analysis was done. Patient 1A had a normal ophthalmic examination and a heterozygous pathogenic variant in the *PDE6B* gene c.1540del PLeu514Trpfs*61. Patients 1B, 2A, and 2B had mid-peripheral retinitis pigmentosa, concentric visual field ring scotomata in both eyes (OU), extinguished electroretinogram (ERG), and homozygous pathogenic variants in the *PDE6B* gene c.1540del PLeu514Trpfs*61. Even though mutations in the *PDE6B* gene usually lead to arRP, they may be inherited in a pseudodominant pattern in geographically isolated populations. Genotyping studies in patients with RP are warranted to classify inheritance mode correctly.

## Introduction

Previous studies have reported that retinitis pigmentosa (RP) is a heterogeneous group of inherited retinal diseases (IRD) characterized by the loss of photoreceptor activity and retinal pigment epithelium function [1]. It affects 1 in 3,000 to 7,000 people, and their symptoms develop from childhood to early adolescence. RP is the most common form of IRD [1]. Although RP is associated with high genetic and clinical heterogeneity, nyctalopia is the most common clinical manifestation. Subsequently, patients may develop progressive loss of their central and peripheral vision [2]. To date, there are 89 causative genes associated with RP [3]. Reports of the phenotypic presentation classically seen in RP were initially found in rodless mice in 1928 [4].

The phosphodiesterase 6B (*PDE6B*) gene is inherited as an autosomal recessive trait and accounts for approximately 8% of all diagnosed autosomal recessive RP (arRP) patients [5]. Khramtsov NV et al. reported that the *PDE6B* gene is located on chromosome 4p16.3. It comprises 22 coding exons and encodes 854 amino acid residues [6]. This gene codes for the beta-subunit of rod-specific cyclic guanosine monophosphate (cGMP) phosphodiesterase 6 (*PDE6*) and is an essential component of the visual phototransduction cascade [7]. To date, 28 mutations in the human *PDE6B* gene have been reported in the literature and classified as leading to the progression of RP [8]. 

We report non-consanguineous patients with pseudodominant inheritance of RP associated with mutations in the *PDE6B* gene.

## Case presentation

**Figure 1:**
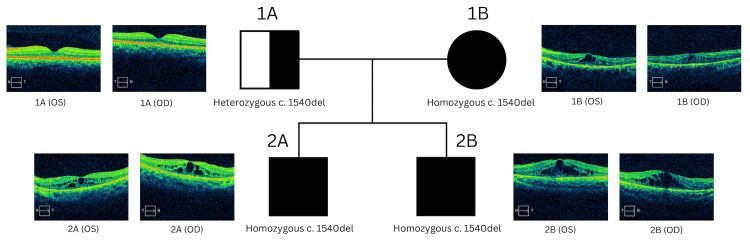
Two-generation pedigree of arRP with PDE6B gene mutation. Pedigree depicts the arRP phenotypes associated with this family's *PDE6B* genetic mutation. It shows the individual mutations in each family member and their corresponding macular optical coherence tomography. arRP: Autosomal recessive retinitis pigmentosa.

Gene sequencing and deletion/duplication analysis using next-generation sequencing (NGS) were utilized to assess the inherited pattern of RP within a non-consanguineous family from a geographically isolated location. Genetic studies were initially done on a child that presented with nyctalopia and his mother, who had a previous diagnosis of RP. The mutational screening was done using a genotyping microarray provided by Invitae Corporation, using NGS technology. Genomic DNA obtained from saliva samples was enriched for targeted regions using a hybridization-based protocol and sequenced using Illumina technology. Following the results, genetic analysis was done on the other two members of the family due to the autosomal recessive inheritance of the disease. The analyses showed a pathogenic homozygous mutation in the PDE6B gene in both children. The mutation is a c.1540del of the variant p.Leu514Trpfs*61. Figure 1 shows this family’s pedigree. The mother presented with the same homozygous mutation as the children, whereas the father had a heterozygous pathogenic mutation in the PDE6B gene. This suggests that, within this family, a variant of this autosomal recessive retinal disease was inherited in a pseudodominant pattern. Each member of this family underwent a comprehensive ophthalmic evaluation.

Patient 1A

A 43-year-old male underwent genetic testing, and analysis showed a heterozygous pathogenic mutation in the PDE6B gene. He had a best-corrected visual acuity of 20/40 and 20/30 +2 in the right eye (OD) and left eye (OS), respectively. Refraction was plano in both eyes (OU). Upon fundus examination, he had normal optic nerves, vessels, and retinal findings. There was no macular edema or mid-peripheral bony spicules OU. Upon macular optical coherence tomography (OCT), the patient had an average macular thickness of 279 µm and 287 µm in OD and OS, respectively. Total macular volume was 10 mm3 and 10.3 mm3 OD and OS, respectively. No visual field was obtained from this patient.

Patient 1B

A 45-year-old female underwent genetic testing, and the analysis showed a pathogenic homozygous mutation in the PDE6B gene. She had been diagnosed with RP at age 26. She has a best-corrected visual acuity of 20/40 +2 and 20/40 +2 in OD and OS, respectively. Refraction was -2.00 +1.50 × 5 and −1.50 +2.50 × 175 in OD and OS, respectively. Upon fundus examination, she had pale optic nerves, attenuated vessels, macular edema (as depicted in Figure 1), and mid-peripheral bony spicules OU. Upon macular OCT, the patient had an average macular thickness of 266 µm and 258 µm in OD and OS, respectively. Total macular volume was 9.5 mm^3^ and 9.3 mm^3^ OD and OS, respectively. Visual field testing revealed a mean deviation of -25.96 dB (p <0.5%) OD and -26.77 dB (p <0.5%) OS. The patient had mild red-green deficiency upon homologous recombination repair (HRR) testing.

Patient 2A 

A 15-year-old boy underwent genetic testing and was subsequently diagnosed with RP. He had presented with progressive worsening visual symptoms (i.e., nyctalopia) and displayed a best-corrected visual acuity of 20/50 +2 and 20/50 +1 in OD and OS, respectively. Refraction was +0.50 DS +2.00 DC × 110 and +0.75 DS +2.00 DC × 95 in OD and OS, respectively. Upon fundus examination, he had pale optic nerves, attenuated vessels, macular edema (as depicted in Figure 1), and mid-peripheral bony spicules OU. Upon macular OCT, the patient had an average macular thickness of 325 µm and 312 µm in OD and OS, respectively. Total macular volume was 11.7 mm^3^ and 11.2 mm^3^ OD and OS, respectively. Visual field testing revealed a mean deviation of -22.28 dB (p <0.5%) OD and -21.03 dB (p < 0.5%) OS. The patient had mild red-green deficiency upon HRR testing.

Patient 2B

A 13-year-old boy underwent genetic testing and was subsequently diagnosed with RP. His uncorrected visual acuity was 20/80 +1 OD and 20/50 +1 OS. Refraction was +2.00 +1.00 × 90 and +3.00 +1.50 DC × 90 in OD and OS, respectively. Upon fundus examination, he had pale optic nerves, attenuated vessels, macular edema (as depicted in Figure 1), and mid-peripheral bony spicules OU. Upon macular OCT, the patient had an average macular thickness of 352 µm and 355 µm in OD and OS, respectively. Total macular volume was 12.7 mm^3^ and 12.8 mm^3^ in OD and OS, respectively. Visual field testing revealed a mean deviation of -19.56 dB (p <0.5%) OD and -16.77 dB (p < 0.5%) OS. The patient had mild red-green deficiency upon HRR testing.

## Discussion

RP is the most common subtype of inherited retinal disease (IRD) [1]. The disease manifests with early-onset nyctalopia followed by visual ﬁeld defects. Literature suggests that RP is inherited in either an autosomal recessive, autosomal dominant, or X-linked pattern in 30%, 20%, and 10% of families, respectively [9]. Nevertheless, mutational screening of the cases we reported on shows a pseudodominant inheritance pattern for the pathogenic variant in the *PDE6B* gene. 

Pseudodominance typically occurs when a patient with a known recessive disorder and a clinically unaffected partner have offspring that are affected with the same recessive disorder as the affected parent [10]. It is usually associated with high mutant frequency in isolated populations. In our family, the affected mother (A2) was homozygous for a pathogenic p.Leu514Trpfs*61 variant on the *PDE6B* gene, whereas the unaffected father (A1) was a heterozygous carrier of the same pathogenic variant. For this reason, they had two affected offspring (B1 and B2) that inherited one variant from each parent. This resulted in patients with the classic arRP phenotype.

Possibilities for pseudodominance were considered. Habibi I et al. showed that autosomal recessive retinal dystrophies could be transmitted in a pseudodominant pattern in consanguineous families [10]. Parents in this family denied consanguinity. However, this family was from the central mountainous region of Puerto Rico, where a high prevalence of this gene mutation may exist. We thus concluded that the presence of this inheritance pattern was a result of geographic isolation.

## Conclusions

Inheritance patterns in patients with RP remain challenging, especially in patients from geographically isolated populations. Genotyping studies in patients with RP are warranted to classify inheritance patterns correctly. If there is more than one affected member of a nuclear family, pseudodominance should be considered. Limitations in this study include the small number of patients. RP is a rare disease as it is, and the frequency of this mutation leading to RP makes it even rarer. Future studies are warranted to find the origin of this gene variant on the island. The focus of such studies should be placed on patients with a clinical diagnosis of RP in the central mountainous regions of Puerto Rico, where consanguinity leads to an increased incidence of autosomal recessive diseases.
